# Validating care and treatment scenarios for measuring decisional conflict regarding future care preferences among older adults

**DOI:** 10.1111/hex.14010

**Published:** 2024-03-07

**Authors:** Craig Sinclair, Ling Yeoh, Ava Karusoo‐Musumeci, Kirsten A. Auret, Josephine M. Clayton, Michelle Hilgeman, Elizabeth Halcomb, Ron Sinclair, Angelita Martini, Anne Meller, Rebecca Walton, Li Wei, Tiet‐Hanh Dao‐Tran, Susan Kurrle, Tracy Comans

**Affiliations:** ^1^ School of Psychology University of New South Wales Sydney New South Wales Australia; ^2^ Neuroscience Research Australia (NeuRA) Sydney New South Wales Australia; ^3^ Rural Clinical School of Western Australia University of Western Australia Albany Western Australia Australia; ^4^ The Palliative Centre, HammondCare Sydney New South Wales Australia; ^5^ Northern Clinical School University of Sydney Sydney New South Wales Australia; ^6^ Tuscaloosa Veterans Affairs Medical Center Tuscaloosa Alabama USA; ^7^ Department of Psychology The University of Alabama Tuscaloosa Alabama USA; ^8^ School of Nursing University of Wollongong Wollongong New South Wales Australia; ^9^ University of Adelaide Adelaide South Australia Australia; ^10^ Brightwater Group Perth Western Australia Australia; ^11^ University of Western Australia Perth Western Australia Australia; ^12^ Prince of Wales Hospital, South Eastern Sydney Local Health District Sydney New South Wales Australia; ^13^ College of Science, Health, Engineering and Education Murdoch University Perth Western Australia Australia; ^14^ Centre for Health Services Research University of Queensland Brisbane Queensland Australia

**Keywords:** advance care planning, decisional conflict, decision‐making, end‐of‐life, scenarios

## Abstract

**Objective:**

Decisional conflict is used increasingly as an outcome measure in advance care planning (ACP) studies. When the Decisional Conflict Scale (DCS) is used in anticipatory decision‐making contexts, the scale is typically tethered to hypothetical scenarios. This study reports preliminary validation data for hypothetical scenarios relating to life‐sustaining treatments and care utilisation to inform their broader use in ACP studies.

**Methods:**

Three hypothetical scenarios were developed by a panel of multidisciplinary researchers, clinicians and community representatives. A convenience sample of 262 older adults were surveyed. Analyses investigated comprehensibility, missing data properties, sample norms, structural, convergent and discriminant validity.

**Results:**

Response characteristics suggested that two of the scenarios had adequate comprehensibility and response spread. Missing response rates were unrelated to demographic characteristics. Predicted associations between DCS scores and anxiety (*r*'s = .31–.37, *p* < .001), and ACP engagement (*r*'s = −.41 to −.37, *p* < .001) indicated convergent validity.

**Conclusion:**

A substantial proportion of older adults reported clinically significant levels of decisional conflict when responding to a range of hypothetical scenarios about care or treatment. Two scenarios showed acceptable comprehensibility and response characteristics. A third scenario may be suitable following further refinement.

**Patient or Public Contribution:**

The scenarios tested here were designed in collaboration with a community representative and were further piloted with two groups of community members with relevant lived experiences; four people with life‐limiting conditions and five current or former care partners.

## 1 INTRODUCTION

Advance care planning (ACP) is a process of reflection, preparation and communication about goals, values and preferences for future care and medical treatment.^1^ Facilitated ACP discussions and documentation of preferences for future care may reduce distress experienced by substitute decision‐makers.^2^, ^3^, ^4^ However the process of arriving at clear and stable preferences has been described by patients and carers as difficult and uncertain, in particular for conditions associated with frailty or gradual and fluctuating decline.^5^, ^6^, ^7^

The concept of decisional conflict was first developed by Janis and Mann,^8^ and has been described as ‘a state of uncertainty about the course of action to take’.^9^ ^,p.25^ Decisional conflict is particularly relevant for complex, high‐stakes decisions involving risk or uncertainty, where there is objectively no clearly superior option. Individuals making such decisions need to understand the risks and benefits of the relevant options and apply their own values, to make a considered decision.^10^, ^11^

Decisional Conflict has been measured using the Decisional Conflict Scale (DCS). The standard format of the DCS contains 16 items with five subscales (informed, values clarity, support, uncertainty and effectiveness), rated on a five‐point Likert scale (strongly agree to strongly disagree) which can be computed to generate an overall score.^9^, ^12^ The DCS has been widely used as a measure of the effectiveness of decision support interventions in healthcare settings.^10^, ^11^ The DCS is typically administered in the context of actual healthcare or treatment decisions which are being contemplated, or have already been made. The nature of the decision being considered and the options available are specified in ‘Part A’ of the DCS, prompting the respondent to select their preferred options. ‘Part B’ contains the standardised questions which address the respondent's self‐reported certainty regarding the decision and the option chosen in Part A.^9^

The DCS is increasingly utilised in studies of ACP and end‐of‐life decision‐making.^13^, ^14^, ^15^, ^16^, ^17^, ^18^, ^19^, ^20^ Evidence of construct validity and association with accepted measures of decision‐making processes have been provided for the DCS Part B scale items.^21^, ^22^ However, existing ACP research has utilised a range of hypothetical scenarios as Part A of the DCS scale, mostly without formal validation or user testing. It stands to reason that the type of decision scenario could influence the psychometric properties of the DCS.^23^ This is particularly relevant for ACP studies, as the medical treatment and/or care scenarios are usually not grounded in actual decisions but instead typically reflect hypothetical scenarios that may be encountered in the future. As shown in Table 1, a scenario might be unsuitable for a range of reasons, including implausibility, incomprehensibility, monotonicity and/or ambiguity.^24^ As DCS scores are increasingly treated as outcome measures in ACP trials, more formal validation of Part A scenarios for the DCS, with normative data enabling comparative studies, will enable greater confidence in the use of the DCS.

**Table 1:** Potential problems with decision‐making scenarios and likely consequences.

Problem	Description	Likely consequence
Implausibility	Scenario is unlikely to occur in real life or not relevant for some or all participants (e.g., a decision about managing a pregnancy for a mixed‐sex sample).	Low engagement among participants, high rates of missing data or ‘I am not sure’ responses, and overall poor ecological validity.
Incomprehensibility	Scenario is difficult to understand due to the presence of medical jargon, inappropriate language, or description of situations that that are difficult for participants to visualise in practical terms.	High rates of missing or ‘unsure’ responses, particularly among participants with lower education or lower health literacy.
Monotonicity	Scenario is presented in a way that strongly favours a particular response.	Ceiling effects on the most popular response, floor effects on the least popular response/s, and a tendency towards very low decisional conflict scores. Low between‐participant variability, resulting in poor discrimination and poor sensitivity to change in response to interventions.
Ambiguity	Scenario presents an ‘impossible’ choice that would likely be difficult for any participant, regardless of personal experience and/or the presence of decision support.	High rates of ‘I am not sure’ responses, platykurtic distribution of decisional conflict scores and poor sensitivity to change in response to interventions.

This study aims to provide preliminary validation data for three hypothetical scenarios relating to life‐sustaining treatments and/or care utilisation for use as Part A scenarios in the DCS. We aim to enable future use of these scenarios in studies of ACP interventions with diverse older adults in community settings.

## 2 METHODS

### 2.1 Care and treatment scenario development

A set of scenarios was developed to reflect a range of possible medical treatment‐ or care‐related decisions relevant to older adults of diverse backgrounds, diagnoses and health states. Wherever possible, the scenarios used lay language, with plain definitions provided for any medical terms. The scenario commenced with an outline of the context (e.g., deterioration during an unplanned hospital admission), an opinion from a relevant health professional or service provider (e.g., doctor), and an indication of the probable outcome, presented in terms of symptom burden, functional impairment and ongoing care needs. For the two medical treatment scenarios, the final sentence clarified that participants should respond based on what they would want to happen, assuming that they were unable to make their own decisions at the time.

The response options for each scenario were for the use of life‐sustaining treatments or care services, adapted from the PREPARE website.^25^ Each scenario included four response options, three of which were graded preferences for different levels of treatment or care involvement (e.g., full treatment with curative intent; trial of active treatment; refusal of active treatment and focus on comfort care) and a fourth which reflected an ‘unsure’ option (see Appendix SA).

Content validity for the scenarios was established through iterative discussions among a panel of clinicians and researchers (the authors of this manuscript). The panel included those with training in geriatrics, palliative medicine, nursing, aged care and psychology, along with a community representative with lived experience as a care partner in end‐of‐life care contexts. The panel developed and refined six scenarios, discussed their clinical plausibility and relevance and selected three scenarios by consensus for further testing in the current study. These scenarios related to decisions about treatments or care in the context of (i) rapid deterioration from infection during an unplanned hospital admission (‘Hospital’), (ii) increasing need for ongoing care in a home setting (‘Care’) and (iii) emergency life‐sustaining treatment after a sudden collapse (‘Emergency’). The scenarios were piloted for plausibility with two groups of community members with relevant lived experiences; four people with life‐limiting conditions and five current or former care partners.

### 2.2 Participants and recruitment

The survey was conducted between August and October 2022 as part of a broader pilot phase for a randomised controlled trial. Older adults (65 years and older) who lived independently and could communicate in English were invited to participate. Convenience sampling methods were used. The survey was publicly advertized through online newsletters (e.g., Advance Care Planning Australia, Centre for Volunteering, Palliative Care Australia) and social media platforms. In addition, one large home care provider organisation distributed an advertisement for the study to approximately 7000 older adult clients receiving community support or home care services. The survey was accessible via a public link to an online REDCap database, with postal, phone or face‐to‐face interview options available. The study procedures were approved by the University of New South Wales Human Ethics Committee (HC220271), and all participants provided informed consent.

### 2.3 Measures

As part of establishing the psychometric properties of the scenarios and response options, the following additional survey measures were also included.

#### 2.3.1 Participant characteristics

Participant age, gender, country of birth, language spoken at home and level of education (school and tertiary). Self‐reported overall health was assessed using a single item from the Short Form Health Survey (SF‐36),^26^ with response options excellent, very good, good, fair and poor. Access to informal care (i.e., family and/or friends) and/or use of formal home care services (i.e., organised care services) were assessed with single items and yes/no response options.

#### 2.3.2 Health literacy

Health literacy was assessed using a three‐item measure, validated for use in primary care and community health settings as a screen for lower health literacy.^27^ The items assessed the frequency (all, most, some, a little, or none of the time) of having assistance in reading medical materials, experiencing difficulty in learning about medical conditions and overall confidence (not at all, a little bit, somewhat, quite a bit, extremely) in filling out medical forms unassisted. Based on a previous study, which showed optimal sensitivity for the detection of practical difficulties in health literacy, we took scores of ‘somewhat’ or less on the single item on overall confidence as criteria for ‘lower health literacy’.^27^

#### 2.3.3 Psychosocial wellbeing

Self‐reported symptoms of anxiety and depression over the past week were assessed using the 14‐item Hospital Anxiety and Depression Scale (HADS).^28^ The HADS is validated among community‐dwelling older adults, yielding a two‐factor model for symptoms of anxiety and depression.^29^ Each dimension is scored with seven items, on a scale from 0 to 21, with higher scores indicating more severe and/or frequent symptoms. In the current study, Cronbach's *α* coefficients were .72 (anxiety) and .82 (depression), respectively.

#### 2.3.4 ACP engagement

The nine‐item Advance Care Planning Engagement (ACP‐9) scale measures self‐efficacy (‘how confident are you that today you could…’) and readiness (‘how ready are you to…’) constructs for different behaviours relevant to ACP (e.g., talking with doctors or loved ones, nominating substitute decision‐makers).^30^ Based on feedback from two panels of people with life‐limiting conditions and current or former care partners, minor modifications were made to the ACP‐9 survey, to better reflect community understandings and local terminology. Specifically, the term ‘medical decision maker’ was rephrased as ‘substitute decision maker for medical decisions’ and ‘if you were very sick and near the end of your life’ was rephrased as ‘if you were very sick and unable to make these decisions for yourself’. Finally, a short introductory statement was added to the ACP‐9 survey, defining the terms ‘advance care planning’ and ‘substitute decision maker’ in the Australian context (see Appendix SB for modified ACP‐9). The summed scale yields scores from 9 to 45, with higher scores indicating higher levels of ACP engagement. In this study, the Cronbach's *α* coefficient was .88.

#### 2.3.5 DCS

The DCS is a validated measure of uncertainty in decision‐making, which can be administered in a number of formats.^9^ In the current study, each of the developed scenarios was presented as Part A followed by a prompt ‘considering the option you chose, please answer the following questions’, and then the Part B DCS questions were presented separately for each of the scenarios. We employed the 10‐item ‘low literacy’ version, which uses a question format, and modified response scale (yes, unsure, no), which can be computed by an algorithm to yield scores between 0 and 100 across four subscales (informed, values clarity, support and uncertainty) and an overall score.^12^

### 2.4 Data analysis

A data analysis plan was prepared and preregistered before analysis (available at https://osf.io/y8dx5). Data analysis was undertaken using R (version 12.6.3) in the R Studio environment (version 1.3.1093).^31^ Missing data patterns were analysed and treated by multiple imputations with chained equations, using the *mice* package, with 10 iterations and 10 imputed datasets. Strip plots and density plots were used to inspect the imputed datasets for plausibility. Pooled parameter estimates were calculated following Rubin's rules, with normality assumed for coefficient estimates (e.g., Pearson's correlation, Cronbach's *α*) given that the sample size was greater than 200.^32^ As a four‐factor structure for the low‐literacy version of the DCS has been established in previous research,^9^, ^12^ the current study used a confirmatory factor analysis approach within a structural equation modelling framework. Factor structure was assessed using the *lavaan* package and diagonally weighted least squares estimation due to the ordinal outcome variables for the DCS.

#### 2.4.1 Comprehensibility

Comprehensibility of the scenarios was assessed by calculating Flesch–Kincaid grade‐level reading scores.^33^ A grade‐level of eight means a reader with an eighth‐grade level of reading would typically be able to understand the text, and this level is typically recommended for text that is targeted at a lay community audience. The number of missing item responses was tabulated for each of the scenarios, among those who had completed the previous scale and proceeded to the section in which the scenarios and DCS questions were administered. To test whether any participant demographic factors were associated with higher missing response rates, Pearson's correlation, *χ* ^2^ or Fisher's exact tests were calculated between missing responses for DCS scale questions and level of education and health literacy.

#### 2.4.2 Sample norms

Proportions, measures of central tendency and measures of dispersion were used to describe sample response characteristics for relevant participant subgroups. As the response options consisted of qualitatively different preferences rather than a Likert‐type scale with presumed linearity, typical standards for classifying floor and ceiling effects were not relevant.^34^ We considered the desired response spread across the four possible options and predefined a scenario as showing floor effects if <10% responses for any single preference option (other than ‘unsure’) were chosen, and ceiling effects if >50% responses for any single preference option (other than ‘unsure’) were chosen.

#### 2.4.3 Structural validity

Structural validity was assessed indirectly by undertaking confirmatory factor analysis for the DCS (Part B) scales accompanying each scenario. Model fit was assessed for the four‐factor solution which was predicted for the 10‐item DCS scale.^9^, ^12^ Criteria for model fit used standard indices (root mean squared error of approximation < 0.05, comparative fit index > 0.9, Tucker–Lewis index > 0.9 and standardised root mean square residual < 0.04).

#### 2.4.4 Convergent and discriminant validity

Convergent validity was assessed by testing bivariate associations between the overall DCS scores for each scenario and self‐reported symptoms of anxiety from the HADS (positive association expected^9^, ^21^) and ACP engagement from the ACP‐9 (negative association expected^35^). Discriminant validity was assessed by testing whether known groups with higher self‐reported health, higher health literacy or actual past experience in ACP discussions with doctors or documenting future care preferences were associated with an increased likelihood of refusing life‐sustaining treatments in response to the care or treatment scenarios, or showing lower overall DCS scores.^7^, ^16^

#### 2.4.5 Internal consistency

Internal consistency for the overall and subscale measures of the DCS were assessed separately for each scenario, using Cronbach's *α*.

## 3 RESULTS

Responses were received from 262 eligible participants, of whom 232 (88.5%) participated online and the remainder by post, phone or face‐to‐face. Participant characteristics (nonimputed demographic data) are shown in Table 2. A majority of participants (136/233, 58.4%) were between 65 and 74 years of age, and 200 (77.5%) were female. The vast majority (253/262, 96.6%) of participants reported speaking English at home, and 180 (69.8%) were born in Australia. Around a quarter of the sample (57/230, 24.8%) reported receiving some assistance at home with activities of daily living; for around a fifth (48/230, 20.9%), this was from a paid service provider (i.e., community aged care organisation).

**Table 2:** Characteristics of participants (nonimputed values).

Participant characteristic		*n* (%)
Age group (year)		
65–74		136/233 (58.4%)
75–84		75/233 (32.1%)
85+		22/233 (9.4%)
Gender		
Male		57/258 (22.1%)
Female		200/258 (77.5%)
Another/prefer not to say		1/258 (0.4%)
Highest level of school education		
Some or all primary school		10/250 (4.0%)
Some secondary school		41/250 (16.4%)
Completed secondary school		199/250 (79.6%)
Highest level of further education		
Certificate (trade, technical or other)		48/246 (19.5%)
Diploma (associate or undergraduate)		19/246 (7.7%)
Bachelor's degree		50/246 (20.3%)
Postgraduate diploma		39/246 (15.8%)
Postgraduate degree (masters or doctorate)		65/246 (26.4%)
None		39/246 (15.8%)
Language spoken at home (English)		253/262 (96.6%)
Born in Australia (yes)		180/258 (69.8%)
Health literacy		
Require help reading medical materials (some of the time or more)		19/257 (7.4%)
Difficult understanding written information (some of the time or more)		18/256 (7.0%)
Confident filling out medical forms alone (somewhat or less)		19/254 (7.5%)
Receives assistance at home		
Has assistance at home (e.g., transport, cleaning, meals, shopping)		57/230 (24.8%)
Has assistance from a paid service provider or organisation		48/230 (20.9%)

*Note*: Denominators reflect the number of raw survey responses received for the relevant question.

### 3.1 Comprehensibility

The Flesch–Kincaid grade reading level scores were 7.8, 7.3 and 11.8 years for the ‘Hospital’, ‘Care’ and ‘Emergency’ scenarios, respectively. Missing response rates averaged 8.18% across all survey items and ranged between 9.9% and 13.2% for the care and treatment scenario questions and DCS items (Table 3). Analysis of missing response rates for the hospital scenario by participant demographic characteristics showed no significant associations for participant age (65–79 vs. 80 years or more; *χ* ^2^ [1, 214] = 1.26, *p* = .26), gender (Fisher's exact test odds ratio = 1.32, *p* = .71), language spoken at home (Fisher's exact test odds ratio = 0.89, *p* = .35), level of education (Fisher's exact test odds ratio = 1.04, *p* = 1.00) or health literacy (Fisher's exact test odds ratio = 1.22, *p* = .59). Similar patterns were observed for the ‘care’ and ‘emergency’ scenarios.

**Table 3:** Grade reading level, missing response patterns and Decisional Conflict Scale scores for the three care and treatment decision scenarios.

	Scenario 1: ‘Hospital’	Scenario 2: ‘Care’	Scenario 3: ‘Emergency’
Flesch–Kincaid grade reading level score	7.8	7.3	11.8
Missing responses (preferences)—*n* (%)	27 (10.3%)	33 (12.6%)	34 (13.0%)
Missing responses (DCS items)—Mean *n* a (%)	25.9 (9.9%)	31.3 (11.9%)	34.7 (13.2%)
DCS overall—Cronbach's *α*	*α* = .94	*α* = .96	*α* = .97
DCS overall—Mean (SD)	31.4 (31.3)	26.9 (31.6)	25.4 (32.3)
DCS ‘Informed’—Cronbach's *α*	*α* = .95	*α* = .96	*α* = .96
DCS ‘Informed’—Mean (SD)	33.4 (36.1)	24.0 (32.9)	29.7 (37.1)
DCS ‘Values Clarity’—Cronbach's *α*	*α* = .93	*α* = .96	*α* = .95
DCS ‘Values Clarity’—Mean (SD)	29.0 (37.0)	22.1 (34.6)	23.2 (35.2)
DCS ‘Support’—Cronbach's *α*	*α* = .79	*α* = .82	*α* = .84
DCS ‘Support’—Mean (SD)	25.4 (30.0)	24.7 (31.0)	23.7 (30.8)
DCS ‘Uncertainty’—Cronbach's *α*	*α* = .92	*α* = .95	*α* = .96
DCS ‘Uncertainty’—Mean (SD)	30.5 (36.9)	24.4 (31.9)	24.0 (36.6)

Abbreviation: DCS, Decisional Conflict Scale.

^a^ For DCS items missing responses are average missing response values from across the 10 items.

### 3.2 Sample norms

**Figure 1:**
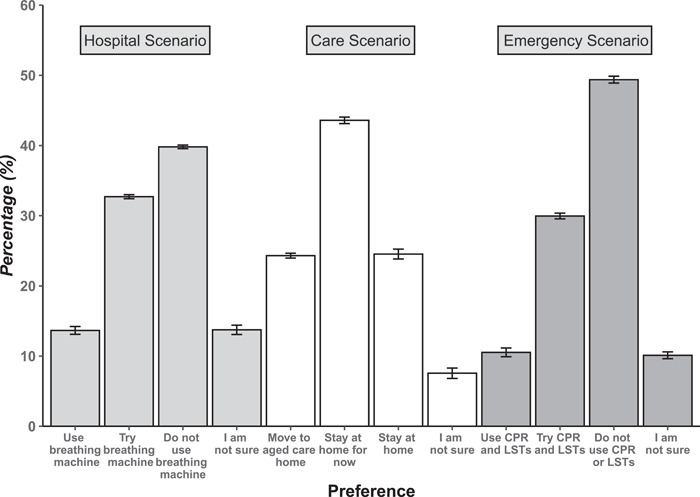
Proportion of participants selecting each of four response options in the three care and treatment scenarios. Figure shows the mean count scores from 10 imputed datasets, with 95% confidence intervals around the point estimate.

The distribution of responses across the three scenarios is shown in Figure 1. For both the ‘hospital’ (39.8%) and ‘emergency’ (49.4%) scenarios, the most commonly selected option was a refusal of treatment. While the ‘hospital’ and ‘care’ scenarios showed acceptable spread across the different response options, the confidence intervals for the point estimates of responses to the ‘emergency’ scenario overlapped the 10% (‘use cardio‐pulmonary resuscitation [CPR]’, floor effect) and 50% (‘do not use CPR’, ceiling effect) thresholds, respectively.

**Figure 2:**
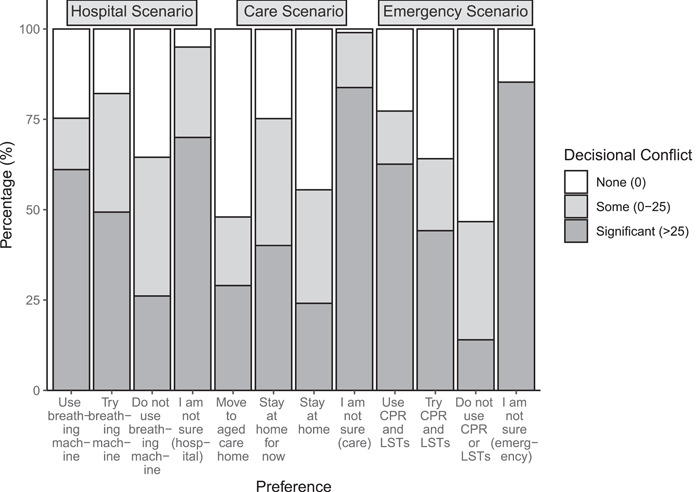
Proportion of participants reporting no, some or significant decisional conflict with respect to the response option selected for each scenario.

The mean DCS overall and subscale scores for the three scenarios are shown in Table 3. A significant subgroup of participants (‘hospital’ = 44.6%; ‘care’ = 36.8%; ‘emergency’ = 35.3%) had DCS overall scores >25, indicating clinically significant decisional conflict.^23^ Participants who selected the ‘I am not sure’ response option had significantly larger DCS overall scores for the ‘hospital’ (*t*[81.7] = 6.28, *p* < .001), ‘care’ (*t*[32.8] = 4.40, *p* < .001), and ‘emergency’ (*t*[54.7] = 6.21, *p* < .001) scenarios. Figure 2 shows the distribution of DCS overall scores for each of the response options.

### 3.3 Structural validity

Confirmatory factor analysis models were separately fitted for the 10‐item DCS scale for each scenario. The predicted four‐factor solution showed acceptable model fit indices for the three care and treatment scenarios (see Table 4).

**Table 4:** Confirmatory factor analysis model results for four‐factor solution of the 10‐item Decisional Conflict Scale, for the three care and treatment scenarios.

	Scenario 1: ‘Hospital’	Scenario 2: ‘Care’	Scenario 3: ‘Emergency’
Model *χ* ^2^ test	239.1	290.9	205.0
Root mean squared error of approximation	0.053	0.059	0.048
Comparative fit index	1.00	1.00	1.00
Tucker–Lewis index	0.99	1.00	1.00
Standardised root mean square residual	0.029	0.027	0.022

### 3.4 Convergent validity

As predicted, overall scores on the DCS for each of the scenarios were positively associated with self‐reported symptoms of anxiety, with pooled Pearson correlation coefficients statistically significant for the ‘hospital’ (*r* = .34, *p* < .001), ‘care’ (*r* = .37, *p* < .001) and ‘emergency’ (*r* = .31, *p* < .001) scenarios. Overall scores on the DCS were also negatively associated with scores on the ACP‐9 scale for the ‘hospital’ (*r* = −.41, *p* < .001), ‘care’ (*r* = −.37, *p* < .001) and ‘emergency’ (*r* = −.40, *p* < .001) scenarios, indicating that higher decisional uncertainty was associated with lower readiness to engage in ACP.

### 3.5 Discriminant validity

Across pooled *χ* ^2^ tests, self‐reported health was not associated with the likelihood of electing the limited or no life‐sustaining treatment options in the ‘hospital’ (*F*[1, 1201.5] = 0.304, *p* = .58) or ‘emergency’ (*F*[1, 5027.1] = 0.03, *p* = .87) scenarios. Similarly, participants categorised as having lower health literacy were not more or less likely to choose these options in either the ‘hospital’ (*F*[1, 2285.6] = 0.018, *p* = .89) or ‘emergency’ (*F*[1, 110.27] = 0.03, *p* = .86) scenarios.

When considering the DCS scale, participants categorised as having lower health literacy had significantly higher overall DCS scores for the ‘hospital’ (*t*[150.3] = 2.61, *p* = .009), ‘care’ (*t*[164.7] = 2.87, *p* = .005) and ‘emergency’ (*t*[124.5] = 15.1, *p* = .046) scenarios. Conversely, those who reported having discussed ACP with their doctor or having completed a written advance care directive had significantly lower overall DCS scores for the ‘hospital’ (*t*[218.3] = 5.75, *p* < .001), ‘care’ (*t*[226.9] = 5.28, *p* < .001) and ‘emergency’ (*t*[230.7] = 6.05, *p* < .001) scenarios. Those who reported receiving formal or informal assistance with activities of daily living at home had somewhat higher overall DCS scores on the ‘care’ scenario than those who did not (33.6 vs. 24.1), although this difference was not statistically significant (*t*[43.7] = −9.48, *p* = .088).

### 3.6 Internal consistency

Internal consistency for the overall DCS, as measured by a pooled Cronbach's *α* coefficient across the imputed datasets, was between 0.94 and 0.97 across the three scenarios. For the DCS subscales, pooled Cronbach's *α* coefficients across the three scenarios ranged between 0.79 and 0.96, indicating high levels of internal consistency (see Table 3).

## 4 DISCUSSION

This study provides preliminary validation data for a set of hypothetical scenarios relating to life‐sustaining treatments and/or care utilisation for use with the DCS in the context of ACP research. Responses from a convenience sample of older adults showed high levels of internal consistency and acceptable performance on a range of measurement criteria, suggesting the suitability of these scenarios in future studies. This study fills a gap in the current literature, as ACP studies using the DCS to date have either used general statements about overall decisional conflict in relation to future healthcare decision‐making,^17^, ^20^ or employed Part A scenarios without reporting formal validation or user testing.

Two of the scenarios (‘hospital’ and ‘care’) showed stronger evidence of optimal comprehensibility and response characteristics. While scenario comprehensibility was not measured through explicit questions to participants, the grade‐level reading scores provided an indicator of the complexity of the written information. The ‘hospital’ and ‘care’ scenarios both scored at less than an eighth‐grade reading level, which we consider acceptable for use among diverse older adults. Other indirect evidence of scenario comprehensibility was the lack of association between missing response rates on these questions and demographic characteristics such as level of education and health literacy. In terms of response spread, the ‘hospital’ and ‘care’ scenarios elicited an adequate spread of responses without ceiling or floor effects. The ‘emergency’ scenario had a higher grade reading level score and elicited a high proportion (>50%) of ‘do not use CPR’ responses. As this ‘emergency’ scenario is considered highly relevant from a clinical perspective, future refinement could simplify the language and adjust scenario characteristics to elicit a broader spread of responses, perhaps by reducing the patient's pre‐existing level of frailty, thus enabling a less pessimistic medical recommendation about the likelihood of successful treatment and recovery.

Consistent with previous research, this study shows a substantial proportion of older adults reported clinically significant levels of decisional conflict when responding to a range of hypothetical care and treatment scenarios.^7^, ^22^ As hypothesised, participants with lower self‐reported health literacy reported higher decisional conflict scores.^7^ Also, as predicted, overall scores on the DCS indicated that higher decisional conflict was associated with increased self‐reported symptoms of anxiety and decreased self‐reported readiness to engage in ACP. The similar magnitude of correlations across the three scenarios suggests that these associations generalise across the range of scenarios tested here. While the cross‐sectional study design limits causal inferences, one plausible explanation is that higher levels of decisional conflict associated with future care and treatment decisions contribute to less readiness to engage in ACP. This is ironic, as previous research has suggested that facilitated ACP interventions are capable of reducing decisional conflict among both patients and family members.^13^, ^14^, ^15^, ^17^, ^18^, ^19^, ^36^, ^37^ Consistent with these findings, participants in the current study who had discussed ACP with their doctor or completed an advance care directive showed lower DCS scores. The relationship between decisional conflict and ACP engagement may also be influenced by other modifiable variables, such as health literacy.^38^ Given the strong association between decisional conflict and experiences of uncertainty,^21^ it is not surprising that the current study found strong associations between DCS scores and symptoms of anxiety.

Although not a focus of the current study, it is noteworthy that in the two medical scenarios (‘hospital’ and ‘emergency’), decisions to ‘use’ or ‘try’ the treatments were associated with higher proportions of participants experiencing significant decisional conflict, as compared to decisions to ‘do not use’ these treatments. Associations between stated preferences and DCS scores have been shown in other healthcare decision‐making contexts,^23^ and response framing effects in advance care directives have also been documented.^39^ Determining whether a particular stated preference in response to a scenario is associated with increased decisional conflict may shed light on participants' decision‐making processes. It may also inform methodological decisions to tether the DCS to specific hypothetical scenarios in ACP research, as opposed to inviting respondents to consider the broader, self‐referential question about decisional conflict regarding their general preferences for future care. While specific scenarios enable more controlled comparisons between participants considering the same hypothetical situation, broader statements, such as _‘_when considering preferences about future care and treatment’^17^, ^20^ may be experienced as more relevant to each participant's unique situation and context. Further investigation of this issue may be a useful topic for future research.

### 4.1 Limitations

Limitations of this study include the relatively small sample size, inability to define a precise response rate, and potential bias towards those with higher health and technology literacy associated with the majority of participant responses being to the online survey. While the authors undertook iterative discussions to optimise the relevance, plausibility and comprehensibility of the scenarios, resource limitations prevented the inclusion of all aspects of content validation described in established frameworks.^40^ Due to the cross‐sectional study design, we were unable to assess test‐retest reliability. Additionally, the sample size was not large enough to enable tests of measurement invariance among sample subgroups on the DCS scale, for example, to determine whether the DCS factor structure was similar for participants with higher versus lower health literacy. The use of hypothetical scenarios about future changes in function or symptom burden associated with changing health states may not always be applicable for people who live with an ongoing disability, and discussions with participants should allow for the person's own interpretation of disability and quality of life.^41^

## 5 CONCLUSION

This study reports on the development of three hypothetical scenarios relating to care and treatment decisions faced by diverse older adults experiencing frailty or diagnosed with life‐limiting conditions. Preliminary validation data support the comprehensibility and validity of two scenarios, with suggestions provided for further development of a third scenario. Participant self‐reported decisional conflict associated with these scenarios was significant and correlated with related constructs in line with prespecified hypotheses. The results should be interpreted cautiously due to the low response rate and potential bias in the study sample. Future studies will validate the use of these scenarios in alternative languages and will explore versions of these scenarios that are appropriate for measuring response preferences and decisional conflict among substitute decision‐makers involved in ACP research.

## AUTHOR CONTRIBUTIONS

**Craig Sinclair**: Conceptualisation; methodology; data curation; supervision; writing—original draft; formal analysis. **Ling Yeoh**: Methodology; data curation; writing—review and editing. **Ava Karusoo‐Musumeci**: Data curation; writing—review and editing. **Kirsten A. Auret**: Conceptualisation; methodology; supervision; writing—review and editing. **Josephine M. Clayton**: Conceptualisation; methodology; supervision; writing—review and editing. **Michelle Hilgeman**: Methodology; writing—review and editing. **Elizabeth Halcomb**: Methodology; writing—review and editing. **Ron Sinclair**: Methodology; writing—review and editing. **Angelita Martini**: Methodology; writing—review and editing. **Anne Meller**: Methodology; writing—review and editing. **Rebecca Walton**: Writing—review and editing. **Li Wei**: Data curation; writing—review and editing; methodology. **Tiet‐Hanh Dao‐Tran**: Writing—review and editing. **Susan Kurrle**: Supervision; methodology; writing—review and editing. **Tracy Comans**: Methodology; writing—review and editing; supervision.

## CONFLICT OF INTEREST STATEMENT

The authors declare no conflicts of interest.

## Supporting information

Supporting information.

Supporting information.

## Data Availability

The analysis plan, scenarios and deidentified data set are publicly available on the project page on Open Science Framework (https://osf.io/sahnf). Translated versions of these scenarios are in development and will be available upon request. Data that support the findings of this study are openly available in the Open Science Framework at https://osf.io/sahnf.
