# Supplementary material for: Validating care and treatment scenarios for measuring decisional conflict regarding future care preferences among older adults
Source: Health Expect. 2024 Mar 7;27(2):e14010. doi: 10.1111/hex.14010 (PMC10918722; doi:10.1111/hex.14010)
Supplement: Supplementary file 1 — Supporting information. [file HEX-27-e14010-s002.docx]

**Appendix A**

English language wording of the ‘Hospital’, ‘Care’ and ‘Emergency’ scenarios and response options.

| **Scenario 1: ‘Hospital’** | |
| --- | --- |
| Imagine you are in hospital and seriously ill from an infection. Despite the treatments, your condition is getting worse quickly. The doctor says that using a breathing machine and other life support treatments might help you survive the infection. But even if you do survive you would probably need ongoing help with breathing from home oxygen equipment. You would be mostly wheelchair bound, and would need a higher level of care at home. If you were in this situation, and unable to make your own decision, what would you want to happen? | USE THE BREATHING MACHINE and any life support treatments that my doctors think might help me survive. I would want to continue life support treatments as long as possible, to give me the best chance of surviving. |
|  | TRY THE BREATHING MACHINE and any life support treatments that my doctors think might help me survive. If after a few days there are no signs of recovery, I would want the doctors to stop these treatments and keep me comfortable. |
|  | DO NOT USE THE BREATHING MACHINE or life support treatments. I would want to be kept comfortable at this stage. I prefer to have a natural death. |
|  | I AM NOT SURE what I would want |
| **Scenario 2: ‘Care’** | |
| Imagine that over time your body has become weaker and more tired. You are no longer able to do jobs around the house, and have had a number of falls with no explanation. Your home care worker is worried you will fall again. It is suggested that you may be safer and get more support in a residential aged care home. A place is available at a nearby aged care home, run by the same organisation as your home care provider. If you were in this situation, what would you want to happen? | STAY AT HOME, even if it means not having extra support close at hand. I would accept the increased risk of getting hurt if I fell again. |
|  | STAY AT HOME FOR NOW, but seriously look at other options for residential aged care in the near future, especially if my condition got any worse. |
|  | MOVE TO THE RESIDENTIAL AGED CARE HOME NOW, while the place is available. |
|  | I AM NOT SURE what I would want. |
| **Scenario 3: ‘Emergency’** | |
| Imagine you have been recovering at home after a period of illness, which has left you weaker than usual. One morning you suddenly collapse and have lost consciousness. Ambulance staff have arrive and are commencing cardio-pulmonary resuscitation (pumping the heart and putting oxygen into your lungs) and are preparing to take you to the hospital. Your doctor previously said that with your current weakness, it is unlikely that resuscitation would be effective. Even if you did survive, you would probably require a long period of recovery in hospital, and would not be able to return to living in your own home. If you were in this situation, and unable to make your own decision, what would you want to happen? | I WOULD WANT CARDIO-PULMONARY RESUSCITATION and any life support treatments that my doctors think might help me survive. I would want to continue life support treatments as long as possible to give me the best chance of surviving. |
|  | START THE RESUSCITATION and any life support treatments that my doctors think might help me survive. If after a few hours, I have not shown signs of recovery, I would want my doctors to cease the treatments and to be kept comfortable. |
|  | DO NOT START THE RESUSCITATION or any life support treatments. I would want to be kept comfortable at this stage. I prefer to have a natural death. |
|  | I AM NOT SURE what I would want. |
