# Supplementary material for: Validating care and treatment scenarios for measuring decisional conflict regarding future care preferences among older adults
Source: Health Expect. 2024 Mar 7;27(2):e14010. doi: 10.1111/hex.14010 (PMC10918722; doi:10.1111/hex.14010)
Supplement: Supplementary file 2 — Supporting information. [file HEX-27-e14010-s001.docx]

**Appendix B**

English version of the Advance Care Planning Engagement Survey (9 item) including original items[30] and modified items for consistency with local jurisdictions in Australia. Boldface is in the table only (not in the administered survey) to emphasize modifications. Response options are as per original items.

|  | Advance Care Planning Engagement Items | Modified Items |
| --- | --- | --- |
|  |  | These questions ask about your feelings to do with advance care planning. Advance care planning means thinking, talking or writing about your wishes for future care, in case you were ever unable to speak for yourself at some time in the future. It may involve appointing a ‘substitute decision maker’ – a person who would make decisions and speak on your behalf about health, medical or lifestyle decisions, if you could not do so for yourself. |
| 8 | How confident are you that today you could ask someone to be your medical decision maker? | How confident are you that today you could ask someone to be your **substitute** decision maker **for medical decisions**? |
| 14 | How ready are you to formally ask someone to be your medical decision maker? | How ready are you to formally ask someone to be your **substitute** decision maker **for medical decisions**? |
| 16 | How ready are you to talk with your doctor about who you want your medical decision maker to be? | How ready are you to talk with your doctor about who you want your **substitute** decision maker **for medical decisions** to be? |
| 20 | How ready are you to sign official papers naming a person or group of people to make medical decisions for you? | How ready are you to sign **a document** naming a person or group of people to make medical decisions for you? |
| 42 | How confident are you that today you could talk with your medical decision maker about the care you would want if you were very sick or near the end of life? | How confident are you that today you could talk with your **substitute** decision maker **for medical decisions** about the care you would want if you were very sick **and unable to make these decisions for yourself**? |
| 43 | How confident are you that today you could talk with your doctor about the care you would want if you were very sick or near the end of life? | How confident are you that today you could talk with your doctor about the care you would want if you were very sick **and unable to make these decisions for yourself**? |
| 48 | How ready are you to talk to your decision maker about the kind of medical care you would want if you were very sick or near the end of life? | How ready are you to talk with your **substitute** decision maker **for medical decisions** about the kind of medical care you would want if you were very sick **and unable to make these decisions for yourself**? |
| 50 | How ready are you to talk to your doctor about the kind of medical care you would want if you were very sick or near the end of life? | How ready are you to talk with your doctor about the kind of medical care you would want if you were very sick **and unable to make these decisions for yourself**? |
| 54 | How ready are you to sign official papers putting your wishes in writing about the kind of medical care you would want if you were very sick or near the end of life? | How ready are you to sign **a document**, putting your wishes in writing about the kind of medical care you would want if you were very sick **and unable to make these decisions for yourself**? |
